# Supplementary material for: Massive compression for high data rate macromolecular crystallography (HDRMX): impact on diffraction data and subsequent structural analysis
Source: J Synchrotron Radiat. 2025 Feb 6;32(Pt 2):385–98. doi: 10.1107/S1600577525000396 (PMC11892891; doi:10.1107/S1600577525000396)
Supplement: Supplementary file 1 [file s-32-00385-sup1.pdf]

JOURNAL OF SYNCHROTRON RADIATION

Volume 32 (2025)

Supporting information for article:

Massive compression for high data rate macromolecular crystallography (HDRMX): impact on diffraction data and subsequent structural analysis

Herbert J. Bernstein, Alexei S. Soares, Kimberly Horvat and Jean Jakoncic

S1. Worked Example of Estimated Costs at AMX

With current beamline performance and upgraded detectors and either assuming very efficient users, or, better, users relying mostly on automated data collection and screening, at AMX, one can achieve 700 samples per day. With each sample generating about 2-5 GB of raw diffraction data per data set and assuming that 2-3 data sets are collected per sample, per standard and per vector, this corresponds to 10 GB per sample, and 7 TB / day. Note that for each sample, using autoProc and fast dp, directories are created in data processing with 0.5 TB of files. Each sample also undergoes rastering with on average 0.5 TB per sample. Subsequent data analysis may generate 0.5 TB of data too.

Only raw diffraction, metadata, data reduction final log and reduced files as well as structure files should be kept along with input and instruction files. Rastering data and intermediate files can be deleted.

This corresponds to up to 11 GB per sample, or 7.7 TB per day and 1.2 PB / year per highly productive and efficient beamline. This is calculated based on 5000 operating hours and 75 % of total time used for user operation.

Improvements in throughput will likely result in double or triple that amount in the next 5 years.

Slow access Amazon Web Services data (Amazon glacier) costs 0.001 cts / GB / month : i.e. per beamline US$1200 per month or US$14,400 per year. This excludes added ingress and egress costs to retrieve and download or upload data.

Assuming that a compression ratio of 1000 can be achieved, one would need to archive 1.2 TB / year. A dual set of RAID1 arrays with a capacity of 4 TB using SSD drives costs ~ US$1500, not counting labor costs.

The more important cost saving is related to facility wide storage: ~ US$200,000/ year for a 4 PB file system maintained for 5 years.

Big question is the computing cost to run these.

S2. Supplementary Tables

Additional compression and structure determination runs were performed for both lysozyme, as shown in Tables S1, S2, S3, S4, and S5, and HIV reverse transcriptase, as shown in Tables S6 and S7. The Table heading arp_100_res_Cycle is the round at which arp built 100 residues or more for lysozyme. The areas of the Tables that are bolded indicate good runs in structure determination. The areas with light blue background required use of heavy atoms from a different compression method or otherwise showed data degradation. The areas with a yellow background indicate runs with serious issues in structure determination.

Table S1 Lysozyme compression runs. The successful runs are bolded.

Extra Compression Ratio	Compression	SHELXD Sol	SHELXE RES	SHELXE FOM/ CONN	DM FOM	ARP res / correct	R	arp_100_ res_Cycle;
**1**	**CBF01 (none)**	**17**	**119**	**0.65/ 0.77**	**0.83**	**127/ 1.00**	**22.4**	**2 (110)**
**25**	**J2K50**	**8**	**111**	**0.65/ 0.76**	**0.83**	**127/ 1.00**	**25.4**	**2 (103)**
**50**	**J2K100**	**4**	**113**	**0.61/ 0.76**	**0.82**	**127/ 1.00**	**23**	**3 (102)**
**100**	**J2K200**	**4**	**104**	**0.65/ 0.76**	**0.82**	**127/ 1.00**	**23.1**	**3 (105)**
251	J2K500<br>J2K500 - HA_solution-J2K200	0<br>NA	0<br>110	0<br>0.64/ 0.76	0<br>0.81	0<br>127/ 1.00	0<br>25.3	5 (101)
498	J2K1000<br>J2K1000 - HA_solution-J2K200	0<br>NA	0<br>59	0<br>0.56/ 0.71	0<br>0.73	0<br>127/ 1.00	0<br>28.9	15 (105)
	J2K1000: 10000trials							
748	J2K1500	NA	NA	NA	NA	NA	NA	
	no HA solution; tried 1 2 and 5k; using HA peaks from jpg200; no success either							
995	J2K2000	NA	NA	NA	NA	NA	NA	
	re-ran:minvalpix=2; forced cell; poor stats							
**8**	**BIN2_ SUM2**	**6**	**109**	**0.63/ 0.76**	**0.83**	**127/ 1.00**	**23.3**	**3 (102)**
**100**	**BIN2_ SUM2_ J2K25**	**16**	**109**	**0.65/ 0.77**	**0.83**	**127/ 1.00**	**23.4**	**2 (111)**
**200**	**BIN2_ SUM2_ J2K50**	**5**	**116**	**0.64/ 0.76**	**0.83**	**127/ 1.00**	**23.4**	**2 (106)**

**399**	**BIN2_ SUM02_ J2K100**	**16**	**106**	**0.64/ 0.76**	**0.82**	**127/ 1.00**	**22.9**	**2 (100)**
**499**	**BIN2_ SUM02_ J2K125**	**8**	**105**	**0.64/ 0.76**	**0.82**	**125/ 1.00**	**24.4**	**2 (101)**

Table S2 Lysozyme compression runs, continued. The successful runs are bolded.

Extra Compression Ratio	Compression	SHELXD Sol	SHELXE RES	SHELXE FOM/ CONN	DM FOM	ARP res / correct	R	arp_100_res_Cycle;
**529**	**BIN2_ SUM02_ J2K130**	**1**	**110**	**0.63/ 0.76**	**0.82**	**127/ 1.00**	**23.6**	**2 (107)**
559	BIN2_ SUM02_ J2K140	NA	NA	0.58/ NA	0.77	NA	22.98	NA
600	BIN2_ SUM02_ J2K150	same destroys all the data						
797	BIN2_ SUM02_ J2K200	18 (CC=10)	no phases			10 res/0.66 con		
	with good peaks: no phases<br>jpeg 200 "destroys" data in ways that phasing does not work with good heavy atoms							
**18**	**HCOMP4**	**10**	**105**	**0.64/ 0.76**	**0.82**	**127/ 1.00**	**24.5**	**2 (103)**
**96**	**HCOMP8**	**30**	**116**	**0.62/ 0.76**	**0.8**	**125/ 1.00**	**25.8**	**2 (104)**
**320**	**HCOMP16**	**0.8 (1/1200)**	**102**	**0.65/ 0.76**	**0.81**	**127/ 1.00**	**23**	**2 (104)**
**335**	**HCOMP20**	**0.8 (1/1200)**	**102**	**0.66/ 0.76**	**0.81**	**127/ 1.00**	**23.9**	**2 (102)**
357	HCOMP24		106	0.62/ 0.77	0.82	127 /0.998	24.2	4 (104)
	0/10000 (usedhcomp08_HA))							
480	HCOMP32	5/5000 (>2500)	117	0.67/ 0.76	0.8	127/ 1.00	22.4	2(115)
672	HCOMP64	0/5000	32	0.56/ 0.74	0.72	5	20.9	NA
**17**	**BIN2_ SUM02_ HCOMP4**	**2**	**106**	**0.65/ 0.76**	**0.83**	**127/ 1.00**	**23.5**	**2 (108)**
**36**	**BIN2_ SUM02_ HCOMP08**	**11**	**109**	**0.60/ 0.76**	**0.82**	**127/ 1.00**	**23.6**	**5 (101)**
**112**	**BIN2_ SUM02_ HCOMP16**	**15**	**103**	**0.63/ 0.76**	**0.82**	**127/ 1.00**	**23.1**	**4 (102)**
**211**	**BIN2_ SUM02_ HCOMP24**	**14**	**99**	**0.61/ 0.76**	**0.82**	**128/ 0.999**	**26.7**	**6 (122)**

Table S3 Lysozyme compression runs, continued. The successful runs are bolded.

Extra Compression Ratio	Compression	SHELXD Sol	SHELXE RES	SHELXE FOM/ CONN	DM FOM	ARP res / correct	R	arp_100_ res_Cycle;
**322**	**BIN2_ SUM02_ HCOMP32**	**10**	**112**	**0.65/ 0.76**	**0.81**	**127/ 1.00**	**23.7**	**3 (102)**
**457**	**BIN2_ SUM02_ HCOMP64**	**1**	**105**	**0.63/ 0.76**	**0.79**	**127/ 1.00**	**24**	**2 (100)**
**10**	**BIN2_ SUM05**	**7**	**109**	**0.66/ 0.77**	**0.84**	**127/ 1.00**	**22.4**	**1 (105)**
**62**	**BIN2_ SUM05_ HCOMP4**	**0.15**	**112**	**0.65/ 0.77**	**0.83**	**127/ 0.999**	**23.5**	**2 (102)**
**220**	**BIN2_ SUM05_ HCOMP16**	**12**	**95**	**0.63/0.76**	**0.82**	**127/ 0.999**	**23.7**	**3 (106)**
**1185**	**BIN2_ SUM05_ HCOMP64**	**5**	**111**	**0.62/0.75**	**0.8**	**126/ 0.999**	**24.2**	**2 (110)**
**20**	**BIN2_ SUM10** **4 / 2000 (1185)**	**0.84**	**108**	**0.64/0.77**	**0.83**	**127 / 1.00**	**22.8**	**3 (108)**
**100**	**BIN2_ SUM10_ HCOMP4** **10 / 10 000 (2329)**	**0.43**	**106**	**0.64/0.76**	**0.83**	**127 / 1.00**	**22.9**	**2 (101)**
**256**	**BIN2_ SUM10_ HCOMP16**	**1**	**115**	**0.63/0.76**	**0.83**	**127 / 1.00**	**23.8**	**2 (112)**
**1336**	**BIN2_ SUM10_ HCOMP64**	**2**	**94**	**0.64/0.75**	**0.79**	**127 / 1.00**	**23.9**	**4 (109)**

Lysozyme data reduction was done in an overall range of 19.72 Å to 1.64 Å with the highest resolution shell being 1.67 Å to 1.64 Å. All data were processed using the same xds.inp that was adjusted for binning and by 2s (pixel size, beam center, oscillation range and masked areas).

Tables S4 and S5 shows the results of the data reduction runs for all lysozyme data sets. Boldface indicates successful runs. A light blue background indicates runs with issues. A light yellow background indicates runs that failed. The Tables highlight the most commonly used parameters (Rmerge, Rpim, completeness, mean I/σ(I), unique reflections) as well as a subsequent set of parameters to illustrate potential damage to data statistics prior to S_SAD phasing (anomalous correlation and mid-slope anomalous probability). For simplification, other parameters usually displayed are not included in this Table. 3600 frames were collected at 7.5 keV to improve Sulfur dF" to maximize likelihood of successful phasing using anomalous signal from the 10 S atoms present in the lysozyme. Note that the protein was crystallized in a NaCl solution resulting in observation of 7 additional peaks that are either Na or Cl ions. The numbers in parentheses are from the highest resolution shell.

Table S4 Lysozyme data reduction runs. The successful runs are bolded. Tables S4 and S5 show the data reduction statistics for all lysozyme data sets.

Compression	Rm all	Rpim all	Mean I/σ(I)	Completeness	Delano correl ½	Mid-slope anom prob	Tot Obs Unique
**native**	**4.1 (7.8)**	**0.9 (6.0)**	**60.9 (8.5)**	**88.9 (19.9)**	**0.427 (0.088)**	**1.842**	**13089 (140)**
**J2K50**	**4.1 (9.8)**	**1.0 (8.6)**	**54.5 (7.6)**	**87.6 (20.3)**	**0.437 (0.126)**	**1.7**	**12901 (154)**
**J2K100**	**4.6 (11.7)**	**1.0 (8.8)**	**50.9 (6.8)**	**87.6 (19.7)**	**0.405 (-0.315)**	**1.593**	**12911 (139)**
**J2K200**	**4.8 (16.2)**	**1.1 (12.4)**	**47.1 (4.5)**	**87.3 (19.7)**	**0.355 (0.178)**	**1.488**	**12859 (139)**
J2K500	5.8 (33.4)	1.3 (25.3)	39.2 (2.0)	87.2 (19.7)	0.276 (0.370)	1.297	12844 (139)
J2K1000	9.9 (47.4)	2.2 (38.0)	20.3 (2.2)	84.5 (17.1)	0.106 (-0.377)	1.103	12475 (121)
J2K1500	24.2 (90.7)	5.5 (73.9)	8.3 (0.7)	87.2 (17.8)	0.043 (0.000)	0.861	12707 (128)
	forcing cell parameters (otherwise failed to converge)						
J2K2000	125.3 (NA)	71.5 (NA)	0.6 (0.7)	45.8 (2.4)	-0.147 (NA)	0.73	6453 (16)
	forcing cell parameters (otherwise failed to converge)						
**BIN2_ SUM02**	**4.6 (7.8)**	**1.0 (6.1)**	**56.5 (8.5)**	**88.7 (18.3)**	**0.378 (0.214)**	**1.758**	**13053 (129)**
**BIN2_ SUM02_ J2K25**	**4.5 (11.6)**	**1.0 (9.2)**	**55.4 (6.4)**	**88.7 (18.3)**	**0.376 (0.073)**	**1.751**	**13059 (129)**
**BIN2_ SUM02_ J2K50**	**4.7 (17.3)**	**1.0 (13.7)**	**52.9 (4.8)**	**88.7 (18.2)**	**0.357 (-0.450)**	**1.644**	**13058 (128)**
**BIN2_ SUM02_ J2K100**	**4.8 (23.4)**	**1.0 (17.8)**	**51.2 (3.9)**	**88.7 (18.3)**	**0.326 (-0.146)**	**1.595**	**13062 (129)**
**BIN2_ SUM02_ J2K125**	**4.8 (28.6)**	**1.0 (21.6)**	**50.7 (2.9)**	**88.7 (18.3)**	**0.341 (0.179)**	**1.553**	**13069 (129)**
**BIN2_ SUM02_ J2K130**	**4.8 (27.8)**	**1.0 (21.3)**	**50.4 (2.9)**	**88.8 (18.3)**	**0.343 (0.285)**	**1.547**	**13070 (129)**
BIN2_ SUM02_ J2K140	169.0 (208.4)	36.3 (175.5)	8.9 (0.6)	88.1 (16.0)	0.007 (-0.478)	0.817	13065 (18)
BIN2_ SUM02_ J2K150	480.3 (-223)	107.3 (-219.6)	2.2 (-0.3)	85.7 (4.0)	-0.092 (NA)	0.716	12262 (32)
BIN2_ SUM02_ J2K200	994.7 (-228.1)	208.8 (-228.1)	1.9 (-0.1)	83.1 (5.6)	-0.042 (NA)	0.765	14201 (44)

**HCOMP4**	**4.6 (9.2)**	**1.0 (6.9)**	**51.7 (7.6)**	**88.8 (19.2)**	**0.365 (-0.066)**	**1.696**	**13081 (135)**
**HCOMP8**	**5.1 (15.4)**	**1.1 (11.9)**	**44.6 (5.6)**	**87.8 (17.0)**	**0.369 (0.770)**	**1.5**	**12911 (119)**
**HCOMP16**	**5.5 (16.3)**	**1.2 (13.6)**	**42.4 (4.8)**	**84.8 (11.6)**	**0.364 (0.000)**	**1.477**	**12448 (81)**
**HCOMP20**	**5.7 (15.9)**	**1.3 (13.0)**	**41.2 (4.7)**	**84.3 (11.1)**	**0.330 (0.000)**	**1.437**	**12366 (78)**

Table S5 Lysozyme data reduction runs, continued. The successful runs are bolded. The numbers in parentheses are from the highest resolution shell.

Compres-sion	Rm all	Rpim all	Mean I/σ(I)	Complete ness	Delano correl ½	Mid-slope anom prob	Tot Obs Unique
HCOMP24	6.0 (15.1)	1.3 (12.7)	40.1 (5.7)	83.1 (9.6)	0.307 (0.000)	1.362	12195 (67)
HCOMP32	6.0 (22.4)	1.3 (19.5)	39.2 (4.5)	79.8 (6.0)	0.254 (0.000)	1.389	11709 (42)
HCOMP64	9.0 (22.4)	2.1 (22.4)	25.5 (3.5)	73.1 (2.3)	0.101 (0.000)	1.248	10717 (1)
**BIN2_ SUM02_ HCOMP4**	**4.6 (8.6)**	**1.0 (6.5)**	**56.0 (7.7)**	**87.9 (18.2)**	**0.361 (0.679)**	**1.721**	**12943 (128)**
**BIN2_ SUM02_ HCOMP8**	**4.5 (9.6)**	**1.0 (7.7)**	**56.2 (8.0)**	**85.9 (18.1)**	**0.303 (0.314)**	**1.773**	**12667 (128)**
**BIN2_ SUM02_ HCOMP16**	**4.5 (14.4)**	**1.0 (12.2)**	**53.4 (6.5)**	**84.9 (14.9)**	**0.384 (0.000)**	**1.653**	**12514 (104)**
**BIN2_ SUM02_ HCOMP24**	**4.9 (11.3)**	**1.1 (9.3)**	**49.7 (5.5)**	**83.2 (14.6)**	**0.318 (0.000)**	**1.512**	**12280 (102)**
**BIN2_ SUM02_ HCOMP32**	**4.7 (18.0)**	**1.0 (15.1)**	**49.4 (5.1)**	**84.1 (12.0)**	**0.359 (0.000)**	**1.558**	**12379 (86)**
**BIN2_ SUM02_ HCOMP64**	**5.1 (18.5)**	**1.1 (15.7)**	**45.4 (6.3)**	**81.8 (5.6)**	**0.288 (0.000)**	**1.467**	**12054 (39)**
**BIN2_ SUM05**	**4.6 (8.2)**	**1.0 (6.4)**	**55.9 (8.1)**	**88.7 (18.3)**	**0.390 (0.109)**	**1.708**	**13055 (129)**
BIN2_ SUM05_ HCOMP4	4.6 (10.2)	1.0 (8.0)	55.1 (7.4)	88.6 (18.3)	0.325 (0.620)	1.703	13049 (129)
BIN2_ SUM05_ HCOMP16	4.6 (10.0)	1.0 (8.2)	53.8 (7.0)	88.3 (17.6)	0.401 (0.232)	1.746	13003 (124)
BIN2_ SUM05_ HCOMP64	5.1 (18.5)	1.1 (18.5)	44.8 (4.0)	86.1 (8.8)	0.311 (0.000)	1.464	12683 (61)
**BIN2_ SUM10**	**4.7 (9.1)**	**1.0 (7.1)**	**53.7 (7.4)**	**88.6 (18.3)**	**0.317 (0.189)**	**1.634**	**13049 (129)**
BIN2_ SUM10_ HCOMP4	4.7 (8.8)	1.0 (6.9)	53.1 (7.1)	88.6 (18.3)	0.279 (-0.072)	1.623	13047 (129)
BIN2_ SUM10_ HCOMP16	4.7 (7.8)	1.0 (6.3)	52.6 (11.5)	88.2 (17.2)	0.336 (0.000)	1.704	12975 (121)

BIN2_ SUM10_ HCOMP64	5.3 (14.8)	1.2 (13.6)	42.3 (4.4)	87.3 (10.2)	0.303 (0.000)	1.424	12850 (71)

S3. HIV Reverse Transcriptase Workflow

We developed a workflow to benchmark the effect of data compression on HIV RT data and on the resulting model (including a large 188 amino acid omit region). The following workflow was used:

1. HIV reverse transcriptase data were compressed and decompressed.
2. HIV reverse transcriptase were reduced using XDS.
3. An initial structure was obtained by the DIMPLE protocol, using a starting model with 188 residues omitted (from Thr^{B}_{240} to GLN^{B}_{428}). The native data set was treated similarly but with no omit region.
4. The resolution of all data was reduced to 2.55 Å. The native data extended to 2.42Å.
5. Structures were refined using REFMAC and PHENIX. A single automated protocol was used for all data refinement.
6. Structure quality statistics were harvested from the atomic coordinate files (Table S8). Note that the large omit region resulted in increased R values.
7. The atomic coordinates resulting from the refinement against the native data (reduced to 2.55 Å) were copied to every directory containing compressed data. Note that this coordinate file did not have an omitted region.
8. The native structure was compared to each compressed data set to generate a real space R value using *overlapmap* (Table S9).
9. The native structure was compared to each compressed data set to generate a real space R value using custom software (Table S10, numbers on the right side).
10. The electron density map obtained using the native structure was then compared to the map obtained using each compressed data set to generate a real space R value using custom software (Table S10, numbers on the left side).
11. The native structure was compared to each compressed data set to generate model to data fit metrics using PHENIX (Table S11).
12. For steps 8 to 11, averages of the quality metrics were separately obtained for Chain A, and for the included and omitted regions of Chain B.

In order to compute real space R-values in a way that (i) permitted the comparisons of maps obtained using non-cognate atomic coordinates and (ii) permitted a custom definition of the real space R-value,

such that the denominator was the variance of the reference map, rather than the total integrated electron density, we apply the algorithm as shown in supplemental Fig. S1.

The data reduction for HIV reverse transcriptase is shown in Tables S6 and S7.

Table S6 HIV reverse transcriptase data reduction runs. The successful runs are bolded. The numbers in parentheses are from the highest resolution shell.

Compres-sion	Rm all	Rpim all	Mean I/σ(I)	Completeness	Delano correl ½
**Native**	**7.9 (144.8)**	**4.1 (72.9)**	**11.4 (1.0)**	**99.9 (100.0)**	**41196 (4602)**
**SUM02**	**8.4 (161.9)**	**4.3 (81.3)**	**11.1 (0.9)**	**99.9 (100.0)**	**41197 (4601)**
**SUM05**	**9.4 (209.4)**	**4.9 (105.1)**	**10.5 (0.7)**	**99.9 (100.0)**	**41217 (4610)**
**SUM10**	**11.1 (269.6)**	**5.8 (136.2)**	**9.3 (0.6)**	**99.8 (100.0)**	**41202 (4613)**
**SUM20**	**15.3 (370.2)**	**8.3 (190.8)**	**7.0 (0.4)**	**99.6 (100.0)**	**41106 (4607)**
**SUM40**	**21.3 (578.4)**	**12.7 (312.9)**	**5.2 (0.3)**	**98.4 (99.9)**	**40527 (4600)**
**BIN2**	**8.7 (135.1)**	**4.5 (67.6)**	**11.2 (1.1)**	**99.8 (100.0)**	**41184 (4605)**
**BIN4**	**12.2 (155.1)**	**6.3 (77.1)**	**9.4 (1.0)**	**99.7 (100.0)**	**41070 (4590)**
BIN6	-6038 (-745)	-3105 (-380)	0 (-0.2)	91.1 (72.7)	37242 (3313)
**J2K10**	**8.0 (147.2)**	**4.1 (74.2)**	**11.4 (1.0)**	**99.9 (100.0)**	**41197 (4601)**
**J2K20**	**9.1 (198.1)**	**4.7 (99.9)**	**9.7 (0.8)**	**99.9 (100.0)**	**41212 (4600)**
**J2K50**	**10.8 (232.4)**	**5.6 (116.8)**	**8.7 (0.7)**	**99.9 (100.0)**	**41210 (4602)**
**J2K75**	**12.3 (265.5)**	**6.3 (133.5)**	**8.8 (0.6)**	**99.9 (100.0)**	**41189 (4597)**
**J2K100**	**13.6 (297.2)**	**7.0 (149.0)**	**8.1 (0.5)**	**99.9 (100.0)**	**41204 (4603)**
**J2K150**	**15.1 (341.0)**	**7.8 (170.5)**	**7.6 (0.4)**	**99.9 (100.0)**	**41173 (4594)**
**J2K200**	**23.8 (169.8)**	**12.3 (87.8)**	**6.0 (1.2)**	**99.9 (100.0)**	**41135 (4598)**
**J2K500**	**27.7 (399.5)**	**14.3 (218.1)**	**4.5 (0.4)**	**99.6 (97.9)**	**40996 (4497)**
**J2K1000**	**28.2 (-4756)**	**14.7 (-2416)**	**3.6 (0.0)**	**96.1 (85.5)**	**39339 (3898)**
J2K2000	81.9 (273.8)	43.4 (162.8)	1.6 (0.4)	95.0 (94.7)	39589 (4428)

Table S7 HIV reverse transcriptase data reduction runs, continued. The successful runs are bolded. The numbers in parentheses are from the highest resolution shell.

Compression	Rm all	Rpim all	Mean I/σ(I)	Completeness	Delano correl ½
**BIN2_ SUM02**	**9.2 (151.1)**	**4.7 (75.7)**	**11.0 (1.0)**	**99.8 (100.0)**	**41164 (4597)**
**BIN2_ SUM02_ J2K50**	**11.7 (273.1)**	**6.0 (137.3)**	**8.1 (0.6)**	**99.9 (100.0)**	**41161 (4586)**
**BIN2_ SUM02_ J2K100**	**15.1 (319.4)**	**7.8 (161.0)**	**7.3 (0.5)**	**99.9 (100.0)**	**40963 (4557)**
**BIN2_ SUM02_ J2K200**	**15.7 (453.1)**	**8.1 (227.7)**	**7.4 (0.3)**	**99.8 (99.9)**	**40978 (4564)**
**BIN2_ SUM02_ J2K500**	**15.4 (689.6)**	**8.2 (347.5)**	**4.6 (4.9)**	**99.0 (96.0)**	**40762 (4412)**
	**forced cell params / fraction 0.2**				
BIN2_ SUM02_ J2K1000	1319 (667)	691.8 (369.2)	0.1 (0.1)	98.8 (97.5)	35814 (4245)
	forced cell params, no refine index; 0.05 indexing				
**HCOMP4**	**9.8 (197)**	**6.9 (125.1)**	**9.5 (0.8)**	**99.9 (100.0)**	**41206 (4596)**
**HCOMP8**	**11.6 (423.6)**	**6.0 (224.2)**	**8.4 (0.2)**	**99.8 (99.9)**	**41178 (4598)**
**HCOMP16**	**10.4 (680.1)**	**5.9 (511.6)**	**6.0 (-0.2)**	**90.8 (69.2)**	**37375 (3422)**
HCOMP24	16.2 (176.9)	9.7 (132.2)	5.5 (0.4)	90.4 (70.0)	37159 (3460)
HCOMP32	9.3 (104.1)	5.2 (102.1)	5.5 (1.0)	66.6 (14.6)	27349 (721)
HCOMP64	14.8 (0.00)	8.4 (0.00)	3.7 (-0.7)	48.3 (1.1)	18190 (52)
**BIN2_ SUM02_ HCOMP04**	**9.7 (166.6)**	**5.0 (83.3)**	**10.6 (0.9)**	**99.9 (100.0)**	**41164 (4596)**
**BIN2_ SUM02_ HCOMP08**	**9.8 (189.4)**	**5.1 (94.6)**	**10.1 (0.8)**	**99.8 (100.0)**	**41169 (4602)**
BIN2_ SUM02_ HCOMP16	12.9 (516.8)	6.6 (260.0)	7.9 (0.3)	99.8 (99.7)	40916 (4549)
	25 % indexing				
BIN2_ SUM02_ HCOMP24	21.2 (644.9)	10.9 (323.1)	6.3 (0.2)	99.9 (99.9)	40880 (4550)
	25 % indexing				

BIN2_ SUM02_ HCOMP32	10.4 (-455.6)	5.6 (-244.3)	8.0 (-0.2)	96.3 (74.3)	39684 (3426)
	25 % indexing				
BIN2_ SUM02_ HCOMP64	9.3 (-169.0)	5.2 (-110.1)	6.4 (-0.5)	90.0 (56.1)	37101 (2583)
	25 % indexing				

Table S8 HIV reverse transcriptase structure quality metrics

DATA	Compr.	Rwork	Rfree	Resol.	Compl.
Native[1]	1	0.2136	0.2752	2.55	99.7
CBF1[2]	1	0.3429	0.4151	2.55	99.78
BIN2_CBF2	8	0.3444	0.4109	2.55	99.69
CBF40	40	0.3426	0.4116	2.55	92.54
BIN2	4	0.3425	0.4124	2.55	99.7
BIN4	16	0.3493	0.4144	2.55	99.37
BIN6	36	0.4216	0.4025	2.56	14.9
J2K200	100	0.362	0.4217	2.55	99.71
J2K500	250	0.3645	0.4294	2.55	95.09
J2K1000	504	0.3823	0.4649	2.55	67.55
J2K2000	1008	0.4545	0.5237	2.55	72.47
BIN2_CBF2_J2K50	200	0.3483	0.4165	2.55	99.57
BIN2_CBF2_J2K200	798	0.3642	0.4189	2.55	96.69
BIN2_CBF2_J2K500	2000	0.3969	0.4707	2.55	75.74
HCOMP04	16	0.342	0.4082	2.55	99.8
HCOMP08	107	0.3547	0.4229	2.55	99.26
HCOMP16	850	0.3874	0.4561	2.55	87.98
HCOMP24	985	0.3741	0.4354	2.55	86.53
HCOMP32	1891	0.4006	0.4925	2.55	64.82
HCOMP64	3037	0.3719	0.4929	2.55	44.15
BIN2_CBF2_HCOMP04	33	0.3434	0.4119	2.55	99.71
BIN2_CBF2_HCOMP08	66	0.3452	0.4145	2.55	99.69
BIN2_CBF2_HCOMP16	253	0.3629	0.4216	2.55	94.18
BIN2_CBF2_HCOMP24	657	0.356	0.418	2.55	93.42
BIN2_CBF2_HCOMP32	1499	0.4024	0.4701	2.55	72.35
BIN2_CBF2_HCOMP64	2999	0.4348	0.5082	2.55	61.2

[1] Native has the entire model in it.

[2] CBF1 has about 30% of the model missing.

Table S9 HIV reverse transcriptase map quality metrics (computed using *overlapmap*). Region A is the entire chain A in the atomic coordinate file. Regions B1, B2, and B3 are three regions in the B chain (significantly, B3 is the omit region that was not present in the phasing model). The *overlapmap* software computes the electron density difference between the comparison map (the map obtained using the compressed data) and the reference map (the map deduced from the model that was refined against the uncompressed data).

DATA	Comp.	CC A	RsRf A	CC B1	RsRf B1	CC B2	RsRf B2	CC B3	RsRf B3
Native	1	0.8706	0.1965	0.8863	0.1919	0.8734	0.1938	0.8852	0.1841
CBF1	1	0.8515	0.2206	0.8699	0.2119	0.8581	0.2172	0.2589	0.4
BIN2_CBF2	8	0.8504	0.2218	0.8686	0.2125	0.8566	0.2197	0.2713	0.401
CBF40	40	0.8228	0.2252	0.8477	0.2188	0.8269	0.2203	0.2338	0.3979
BIN2	4	0.8518	0.2217	0.8705	0.2127	0.8581	0.2188	0.2731	0.4004
BIN4	16	0.8497	0.2226	0.8648	0.2159	0.8511	0.2223	0.3034	0.403
BIN6	36	0.3388	0.3449	0.3263	0.275	0.3168	0.3891	0.074	0.4544
J2K200	100	0.8541	0.2204	0.8729	0.2144	0.8598	0.2193	0.3643	0.4017
J2K500	250	0.8401	0.221	0.8581	0.216	0.8484	0.2199	0.2698	0.4006
J2K1000	504	0.8304	0.2202	0.8473	0.2156	0.8403	0.2205	0.2099	0.399
J2K2000	1008	0.8367	0.2127	0.8564	0.2093	0.8522	0.2133	0.3212	0.3945
BIN2_CBF2_J2K50	200	0.8444	0.2224	0.8635	0.2121	0.8507	0.2204	0.2635	0.4022
BIN2_CBF2_J2K200	798	0.8296	0.2238	0.8464	0.2172	0.8358	0.2231	0.2614	0.4023
BIN2_CBF2_J2K500	2000	0.8168	0.2227	0.8312	0.2175	0.8246	0.2222	0.1723	0.4047
HCOMP04	16	0.853	0.2208	0.8709	0.2127	0.8595	0.2169	0.2659	0.3999
HCOMP08	107	0.8445	0.2213	0.8613	0.2149	0.8512	0.2207	0.2552	0.3987
HCOMP16	850	0.8227	0.2214	0.84	0.2143	0.8366	0.2193	0.1704	0.4005
HCOMP24	985	0.7841	0.2358	0.8024	0.2248	0.8006	0.231	0.1495	0.403
HCOMP32	1891	0.8114	0.222	0.8273	0.2158	0.8279	0.2189	0.1849	0.3962
HCOMP64	3037	0.8035	0.221	0.8177	0.2173	0.819	0.2193	0.2077	0.3883
BIN2_CBF2_HCOMP04	33	0.8508	0.2218	0.8683	0.2127	0.8556	0.2195	0.2755	0.401
BIN2_CBF2_HCOMP08	66	0.8491	0.2218	0.8667	0.2128	0.8546	0.2194	0.2682	0.4015
BIN2_CBF2_HCOMP16	253	0.8338	0.2235	0.8514	0.215	0.8434	0.2204	0.2435	0.4027
BIN2_CBF2_HCOMP24	657	0.8346	0.2238	0.8543	0.2178	0.8428	0.2219	0.2817	0.4021
BIN2_CBF2_HCOMP32	1499	0.8211	0.2232	0.8355	0.2169	0.8337	0.221	0.1921	0.4022
BIN2_CBF2_HCOMP64	2999	0.8116	0.2222	0.8282	0.2152	0.825	0.2197	0.1625	0.4016

Table S10 HIV reverse transcriptase map quality metrics (computed using *custom software*). Chain A is the entire chain A in the atomic coordinate file. Chains B1, B2, and B3 are three chains in the B chain (significantly, B3 is the omit region that was not present in the phasing model). In this software, the real space R value is computed in the envelope of each amino acid by adding the total differences between a comparison map and a reference map, divided by the R.M.S. variance of the reference map. Two columns are shown for each chain. In both columns, the comparison map is the map obtained using the compressed data. In the left column, the reference map is the map obtained using the uncompressed data. In the right column, the reference map is the map deduced from the model that was refined against the uncompressed data.

DATA	Comp	Chain A		ChainB1		ChainB2		ChainB3	
Native	1	0.003	0.152	0.003	0.151	0.003	0.155	0.003	0.146
SUM1	1	0.187	0.231	0.187	0.227	0.188	0.233	0.329	0.323
BIN2_SUM2	8	0.185	0.229	0.185	0.226	0.185	0.23	0.328	0.321
SUM40	40	0.208	0.249	0.212	0.251	0.214	0.25	0.338	0.329
BIN2	4	0.184	0.228	0.184	0.224	0.185	0.23	0.329	0.323
BIN4	16	0.186	0.226	0.188	0.223	0.19	0.229	0.33	0.324
BIN6	36	0.423	0.421	0.407	0.403	0.404	0.426	0.436	0.391
J2K200	100	0.192	0.252	0.194	0.247	0.197	0.263	0.34	0.341
J2K500	250	0.204	0.243	0.21	0.24	0.206	0.247	0.332	0.329
J2K1000	504	0.227	0.255	0.228	0.252	0.225	0.257	0.331	0.326
J2K2000	1008	0.24	0.349	0.241	0.335	0.232	0.356	0.344	0.36
BIN2_SUM2_J2K50	200	0.19	0.237	0.191	0.237	0.192	0.239	0.329	0.324
BIN2_SUM2_J2K200	798	0.19	0.243	0.192	0.246	0.191	0.243	0.326	0.322
BIN2_SUM2_J2K500	2000	0.212	0.269	0.21	0.271	0.209	0.265	0.325	0.321
HCOMP04	16	0.185	0.229	0.187	0.226	0.187	0.231	0.33	0.325
HCOMP08	107	0.189	0.245	0.193	0.247	0.189	0.245	0.33	0.327
HCOMP16	850	0.214	0.256	0.218	0.261	0.216	0.253	0.325	0.319
HCOMP24	985	0.42	0.399	0.417	0.395	0.417	0.4	0.399	0.386
HCOMP32	1891	0.247	0.27	0.243	0.267	0.247	0.276	0.325	0.32
HCOMP64	3037	0.274	0.304	0.274	0.301	0.27	0.309	0.327	0.331
BIN2_SUM2_HCOMP04	33	0.184	0.228	0.184	0.224	0.185	0.229	0.329	0.322
BIN2_SUM2_HCOMP08	66	0.186	0.231	0.186	0.228	0.189	0.233	0.328	0.322
BIN2_SUM2_HCOMP16	253	0.19	0.255	0.19	0.259	0.193	0.255	0.325	0.323
BIN2_SUM2_HCOMP24	657	0.192	0.238	0.196	0.241	0.196	0.24	0.328	0.325
BIN2_SUM2_HCOMP32	1499	0.204	0.284	0.205	0.293	0.201	0.28	0.324	0.322
BIN2_SUM2_HCOMP64	2999	0.228	0.268	0.224	0.269	0.228	0.264	0.321	0.315

Table S11 HIV reverse transcriptase map quality metrics (compared using PHENIX). MapCC is the map correlation coefficient, AAflp are the number of amino acids where the side chain may be in an incorrect conformation, and PoorD is the number of amino acids where the side chain has no conformation that has a good agreement between the model and the data.

DATA	Comp	Rwork	MapCC	AAflp	PoorD
Native	1	0.2106	0.9184	139	85
SUM1	1	0.2655	0.8665	163	109
BIN2_SUM2	8	0.2758	0.863	164	110
SUM40	40	0.2818	0.8375	174	120
BIN2	4	0.2665	0.8705	162	108
BIN4	16	0.2688	0.8749	164	110
BIN6	36	0.4909	0.5897	198	144
J2K200	100	0.2633	0.8717	166	112
J2K500	250	0.2787	0.8453	179	125
J2K1000	504	0.2952	0.846	201	147
J2K2000	1008	0.3496	0.8152	235	181
BIN2_SUM2_J2K50	200	0.2782	0.8645	155	101
BIN2_SUM2_J2K200	798	0.2715	0.8475	155	101
BIN2_SUM2_J2K500	2000	0.2956	0.8077	185	131
HCOMP04	16	0.271	0.868	162	108
HCOMP08	107	0.2774	0.8635	165	111
HCOMP16	850	0.2921	0.8211	197	143
HCOMP24	985	0.2921	0.8211	197	143
HCOMP32	1891	0.332	0.804	227	173
HCOMP64	3037	0.3682	0.781	291	237
BIN2_SUM2_HCOMP04	33	0.2714	0.8644	163	109
BIN2_SUM2_HCOMP08	66	0.2704	0.8661	161	107
BIN2_SUM2_HCOMP16	253	0.2627	0.8594	166	112
BIN2_SUM2_HCOMP24	657	0.2627	0.8594	166	112
BIN2_SUM2_HCOMP32	1499	0.297	0.8084	186	132
BIN2_SUM2_HCOMP64	2999	0.2987	0.7867	208	154

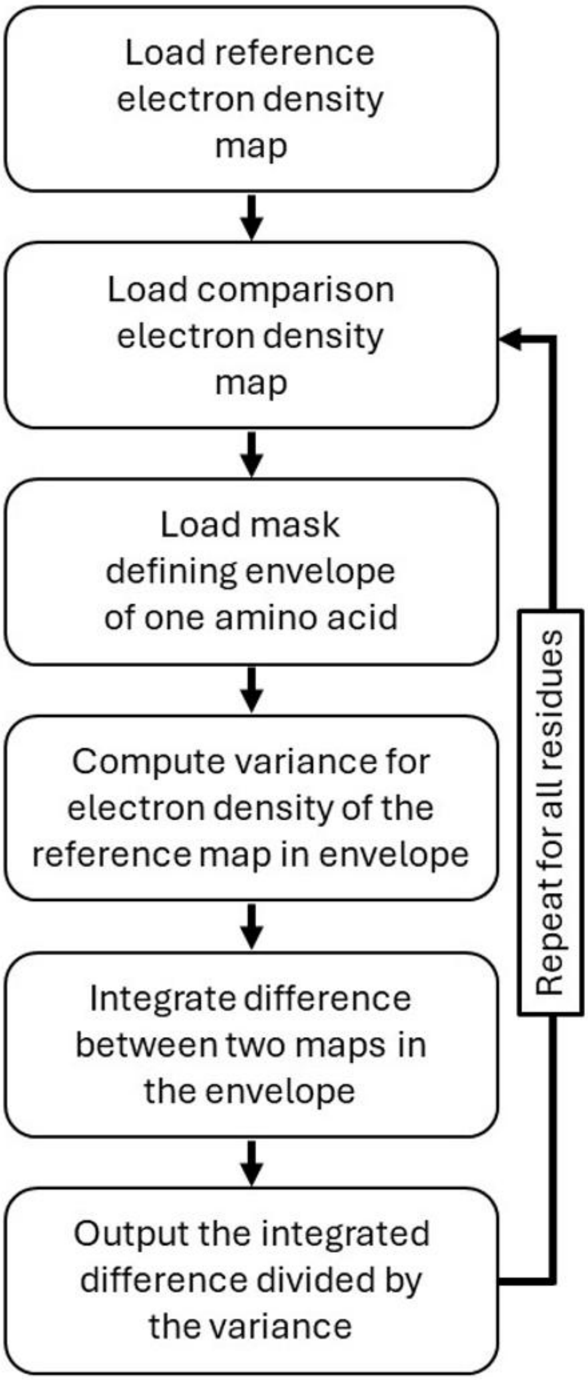

Figure S1 Custom software was prepared to compute real space R-values in a way that (i) permitted the comparisons of maps obtained using non-cognate atomic coordinates and (ii) permitted a custom definition of the real space R-value, such that the denominator was the variance of the reference map (rather than the total integrated electron density).
